# Floral Nectary Anatomy and Ultrastructure in Mycoheterotrophic Plant, *Epipogium aphyllum* Sw. (Orchidaceae)

**DOI:** 10.1155/2015/201702

**Published:** 2015-03-25

**Authors:** Emilia Święczkowska, Agnieszka K. Kowalkowska

**Affiliations:** ^1^Department of Plant Taxonomy and Nature Conservation, University of Gdansk, Wita Stwosza 59, 80-308 Gdańsk, Poland; ^2^Department of Plant Cytology and Embryology, University of Gdansk, Wita Stwosza 59, 80-308 Gdańsk, Poland

## Abstract

*Epipogium aphyllum* is a European-Asian obligatory mycoheterotrophic orchid containing no chlorophyll. Flowers are not resupinate with a sack-shape spur and cordate lip, which is divided into two parts: the basal (hypochile) and distal one (epichile). The floral analysis provides strong evidence to conclude that nectar is secreted on the upper surface of pink-coloured papillate ridges and epidermal (adaxial) cells at different place in spur, especially at the apex. The exudation on papillae has been observed through the entire anthesis and it has been stained on polysaccharides, proteins, and lipids. The dense cytoplasm of papillae contains profuse endoplasmic reticulum, plentiful vesicles (bigger ones with tannin-like materials), numerous mitochondria, sometimes dictyosomes, starch grains, and plastids with tubular structures. The large electron-dense bodies in cell walls are structurally the same as tannin-like materials from vesicles that are in contact with plasmalemma. The rupture of thin layer of swelled cuticle is caused by pressure of gathered substances exuded due to granulocrine secretion. The idioblasts with raphides occur mainly in tepals tissue. The dynamic changes of the nectar exudation, released through endocrine secretion, have been noticeable during the anthesis: both on the lip and inside the spur. The nectar secretion is not dependent on the colour form of *E. aphyllum* blooming shoots. The floral biology and ultrastructure differ from mycoheterotrophic plants known up to date.

## 1. Introduction

The nectar secretion and scent production in orchid flowers can be divided into three groups. The species from the first one, for example,* Anacamptis*,* Gymnadenia*, and* Platanthera*, produce the nectar and emit a strong scent. Another group, for example,* Neottia, Coeloglossum,* and frequently* Epipactis*, is characterized by the production of large amounts of nectar without scent emission. Other species, for example,* Dactylorhiza *and* Orchis*, emit delicate scent, but do not produce nectar and their floral appearance often mimics nectariferous plants [1]. The investigated orchid species,* Epipogium aphyllum* (Ghost Orchid), is a very rare, terrestrial obligatory mycoheterotrophic orchid containing no chlorophyll. It is known for its intermittent (like a “ghost”) appearance. It belongs to the first group: it produces nectar and emits scent, most frequently defined as the vanilla fragrance [2–4] and sometimes as the banana one [1, 2]. The plant growth is rapid. The stem measures from 2.5 to 35 cm high and it is whitish to yellow with pinkish-brown tinge, short, pale rose-colored dashes. Sporadically, the albino blooming shoots occur [5]. There are 2-3 brownish scales at the base of stem. The inflorescence is lax, 1 to 6(8) flowers about 1.5–3 cm in vertical diameter, pendant, with small pedicel, and not resupinate. Petals and sepals are of the same shape and are yellowish, lanceolate, and obtuse, facing downwards, 1.3–1.7 cm long, and 2–4.5 cm wide. Petals are two times wider than the sepals [1, 4, 6]. The lip is divided into two parts: distal epichile and proximal hypochile. The epichile is cordate, acute with a furrow in the middle, whitish with pink tinge inside, and four pink or purple-coloured papillate ridges. The hypochile is short with two rounded, raised lobes. The sharply bent backwards lip is ended with sack-shape spur. The whitish spur has a pink pattern inside, rounded at the end and bended up [1, 2, 5]. The flowers open a few days after the appearance of the ground primordium and bloom for few days only. After this time, the capsules are formed [5]. Blooming shoots persist for about 1.5 weeks and then fade [2]. The maximum floral duration on the shoot is up to 8 days (Święczkowska, in prep.). The pollinators of Ghost Orchid flower are guided towards the secretion in spur by pink ridges present on the lip. According to Rohrbach [7], the “yellow zone,” part between a hypochile and a spur, secretes a sugary fluid, which may be the nectar. Claessens and Kleynen [2] found a little amount of nectar in the spur. These authors and also Godfery [8] claim that the lip may be a nectar-secreting organ. The presence of structures such as papillae and keels suggests that reputedly sugary fluids can be produced there, which is an incentive for pollinators. Delforge [1] claims that the two short lateral lobes border a broad nectariferous cup, which is prolonged into a spur, whereas Baumann et al. [6] believe that flowers of* Epipogium aphyllum *only imitate flowers of nectariferous plants. 

The aims of this study were

1. the anatomical confirmation of nectar secretion in the lip, as well as inside the spur of the* Epipogium aphyllum* flower;
2. the examination of that whether the presence of nectar is related to the various, colorful forms of blooming shoots.

## 2. Materials and Methods

Floral material for the study was collected from the largest population of* Epipogium aphyllum* in Poland (above 300 blooming shoots, regularly blooming every year), which is located in Darżlubska Forest, near Wejherowo, in the Pobrzeże Kaszubskie region (northern Poland) [9]. The population is growing at anthropogenic habitat of* Luzulo-Fagetum* beech forests (*Luzulo Pilosae-Fagetum*) with modified emission of dust from a nearby cement plant and the artificial introduction of pine to tree stand. The collection of material for the presented research was conducted under a permit from the Regional Director for Environmental Protection in Gdańsk, number RDOŚ-Gd-PNII.6400.3.2013.MaK.1. In the Polish Red Book of Plants, the Ghost Orchid is placed as critically endangered with extinction (CR category) [10].

**Figure 1:**
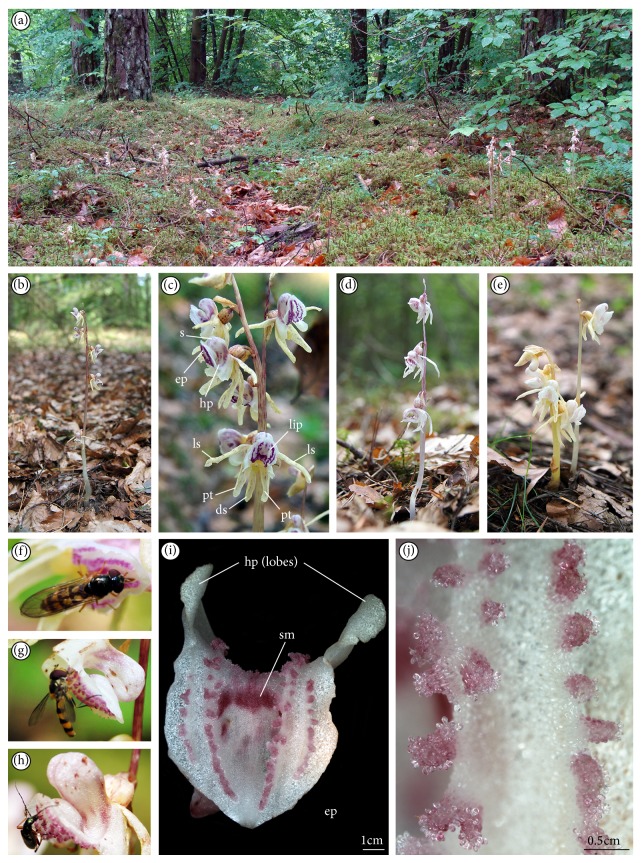
*Epipogium aphyllum*: (a) habitat in Darżlubska Forest, (b) normal blooming shoot, (c) inflorescence of normal blooming shoot, (d) half-albino blooming shoot (without yellow pigment), and (e) half-albino blooming shoot (without pink pigment). Visiting insects licking the fluids on the lip: (f, g)* Meliscaeva cinctella* and (h)* Chrysanthia geniculata*. (i) Lip epichile with four pink papillate ridges leading to the upwardly located spur and central furrow with pink smudge (*sm*); hypochile with two lateral lobes (LM). (j) Multicellular outgrowths on ridges consisted of groups of rounded papillae (LM) (ds: dorsal sepal, ls: lateral sepal, pt: petal, lip with hp–hypochile and ep–epichile, s: spur, and sm; pink smudge on epichile).

Flowers of a typical form (Figure 1(a)) were collected in July and August 2013. For comparison, there were also collected flowers of the half-albino type (without yellow colour) and another type of albino (without pink colour). Flowers were collected, respectively, on the 2nd, 3rd, 4th, 5th, 6th, and 8th days after the opening of a flower, in triplicate.

Fresh flowers were observed under a Nikon SMZ1500 stereomicroscope. Pieces of tepals and labellum tissue were fixed in 2.5% glutaraldehyde (GA) in 0.05 M cacodylate buffer (pH = 7.0). The material for light microscopy (LM) was rinsed with cacodylate buffer and then dehydrated. The dehydrated material was embedded in epoxy resin [11] and methylmethacrylate-based resin (Technovit 7100, Heraeus Kulzer GmbH). Sections (1–5 *μ*m thick) were cut with metal knives, respectively, and mounted on glass slides. For LM, the material was stained with 0.05% Toluidine Blue O (TBO) for 1 min at 60°C on a hot plate [12, 13]. Aniline Blue Black (ABB, C.I. 20470) was used for detection of water-insoluble proteins [14]. The PAS reaction was used to identify the presence of water-insoluble polysaccharides [14] and Sudan Black B (SBB) was used for lipid localization [15]. Auramine O was used for detection of cuticle layer and also for the presence of lipids [16]. For scanning electron microscopy (SEM), after dehydration in an ethanol series, the samples were dried by the critical point method using liquid CO_2_, coated with gold and observed in a Philips XL-30 in the Laboratory of Electron Microscopy of the University of Gdańsk. For transmission electron microscopy (TEM), the floral material was fixed in 2.5% GA in 0.05 M cacodylate buffer (pH 7.0). The material was post-fixed overnight in 1% OsO4 in cacodylate buffer in a refrigerator and then rinsed in the buffer. After 1 hr in 1% uranyl acetate in distilled water, the material was dehydrated with acetone and embedded in Spurr's resin. Ultrathin sections were cut on a Sorvall MT 2B ultramicrotome with a diamond knife and contrasted with uranyl acetate and lead citrate. The sections were examined in a Philips CM 100 transmission electron microscope in the Laboratory of Electron Microscopy of the University of Gdańsk. Samples were prepared in accordance with procedures described elsewhere [17–20].

The preparations (TBO, ABB, PAS, and SBB) were examined and photographed with a Nikon Eclipse E 800 light microscope equipped with a Nikon DS-5Mc camera and analyzed with Lucia Image software. The preparations from Auramine O staining were observed in the above equipment with block of filters: B-2A (EX 450–490 nm, DM 505 nm, BA 520 nm).

## 3. Results

Three forms of blooming shoots according to different pigments were distinguished in the Darżlubska Forest population (Figure 1(a)). The whitish to yellow stem, or pale brown with rose-colored dashes, 2.5–35 cm high with 1–6 flowers, was defined as normal commonly known form of blooming shoot (Figures 1(b) and 1(c)). The second form was half-albino type without yellow pigment (Figure 1(d)), 6–22 cm high with 1–5 flowers. The stem was often fragile and sometimes twisted. The third form without pink pigment was also half-albino (Figure 1(e)), 10–16 cm high with 1–4 flowers. The insects visiting flowers (not pollinators) and licking the fluid on papillate ridges were mainly* Meliscaeva cinctella* (Figures 1(f) and 1(g)) and rarely beetles, for example,* Chrysanthia geniculata* (Figure 1(h)). There were no differences among anatomical cross-sections of different flower forms (not illustrated).

**Figure 2:**
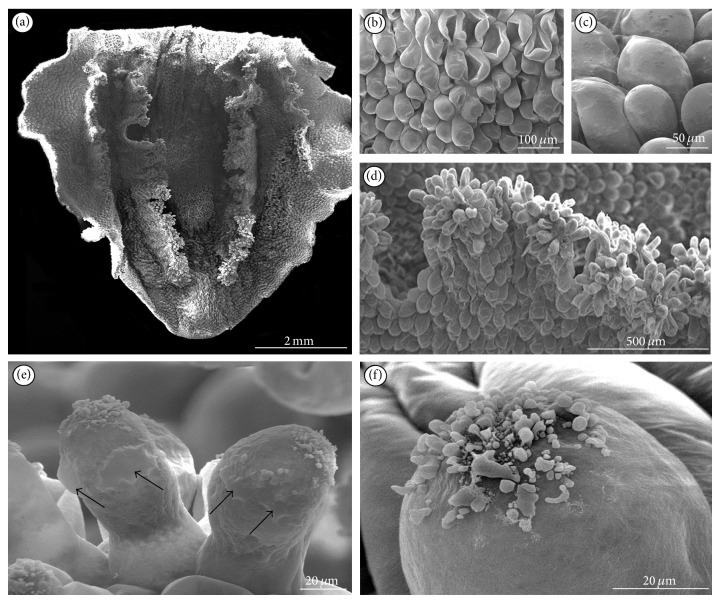
SEM studies of lip on the 8th day of anthesis. (a) Cordate epichile with papillate ridges. (b) Rounded (often sunken) cells from the furrow. (c) Rounded cells close to margin without secretions. (d) Papillate ridges with multicellular outgrowths. (e) Papillae with the cuticle swellings (*arrows*) at their backsides and secretion at their apices. (f) Secretion at the apex of papilla.

On the normal type of blooming shoots (Figure 1(c)), the lip consisted of two parts: basal hypochile and apical epichile (Figure 1(i)). The hypochile formed by the two short lateral lobes (Figure 1(i)) was prolonged into a sack-shape spur (Figure 1(c)) and relative to the epichile was set at right angle. The epichile was cordate, acute with irregular margins (Figures 1(i) and 2(a)). In contrast to other orchid species,* Epipogium aphyllum *possessed central furrow with pink smudge in epichile (Figure 1(i)), not in hypochile. On both sides, the furrow was surrounded by two pink papillate ridges leading to the upwardly located spur (Figures 1(c) and 1(i)). The papillate ridges (Figures 1(i) and 2(a)) were multicellular outgrowths with groups of rounded papillae at their apices (Figures 1(j) and 2(d)). The exudation was only present on the upper surface of papillae (Figures 2(d), 1(e), and 1(f)) through the whole flowering period. The nectar secretion was not recorded in the furrow of epichile (Figure 2(b)) or on other lip cells (Figure 2(c)). The most abundant secretion was observed on the 8th day of anthesis (Figures 2(e) and 2(f)). In some papillae, at their backsides, the cuticle swellings were observed (Figure 2(e)). Nevertheless, the flowers were collected on the 2nd, 3rd, 4th, 5th, 6th, and 8th days of anthesis, and the amounts of nectar presented on papillae were various. Comparing the results from collected flowers, we could draw a conclusion that the quantity of secretion from labellar papillae and spur showed some increasing tendency beginning from the 2nd day up to the 4th day of anthesis. There was decrease in the amount of nectar on the 5th day. Then, on the 6th day, the secreted nectar was increased up to the end of anthesis (the 8th day), where the highest quantity of secretory substances was observed in both the lip and the spur. The most intensive secretion was recorded at average daily temperatures from 17.1°C to 19.9°C. Above this temperature, only a small amount of secretion on the upper surface of papillae was noted.

**Figure 3:**
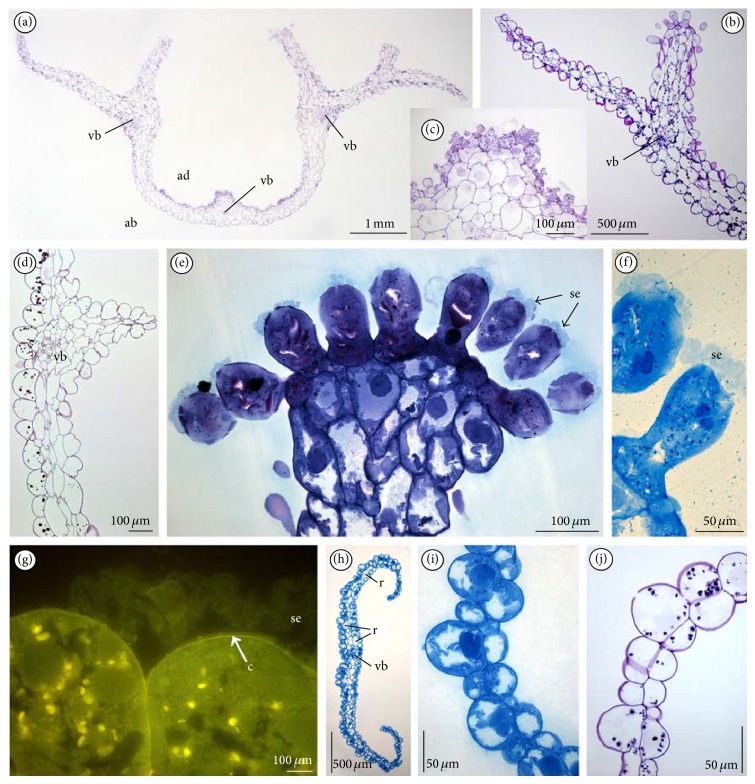
LM observations of tepals. (a) Cross-section of lip with collateral vascular bundles in parenchyma (PAS). (b) Lip part with starch grains in cells on the 3rd day of anthesis stained for polysaccharides insoluble in water (PAS). (c) Central part of furrow from the lip with sunken cells on the top, 3rd day, stained for polysaccharides (PAS). (d) Lip part with starch grains only in abaxial epidermis, 6th day (PAS). (e) Papillate ridges, note the secretion at the top and dense cytoplasm in papillae, the 4th day of anthesis (TBO). (f) Secretion on papillae, 4th day, stained for proteins (ABB). (g) Thin layer of cuticle, 6th day (Auramina O). (h) Cross-section of dorsal sepal with idioblasts with raphides (TBO). (i) Dorsal sepal stained for proteins (ABB). (j) Dorsal sepal with tiny starch grains (PAS) (ad: adaxial (inner) surface, ab: abaxial (outer) surface, c: cuticle, r: raphides, se: secretion, and vb: vascular bundle).

**Figure 4:**
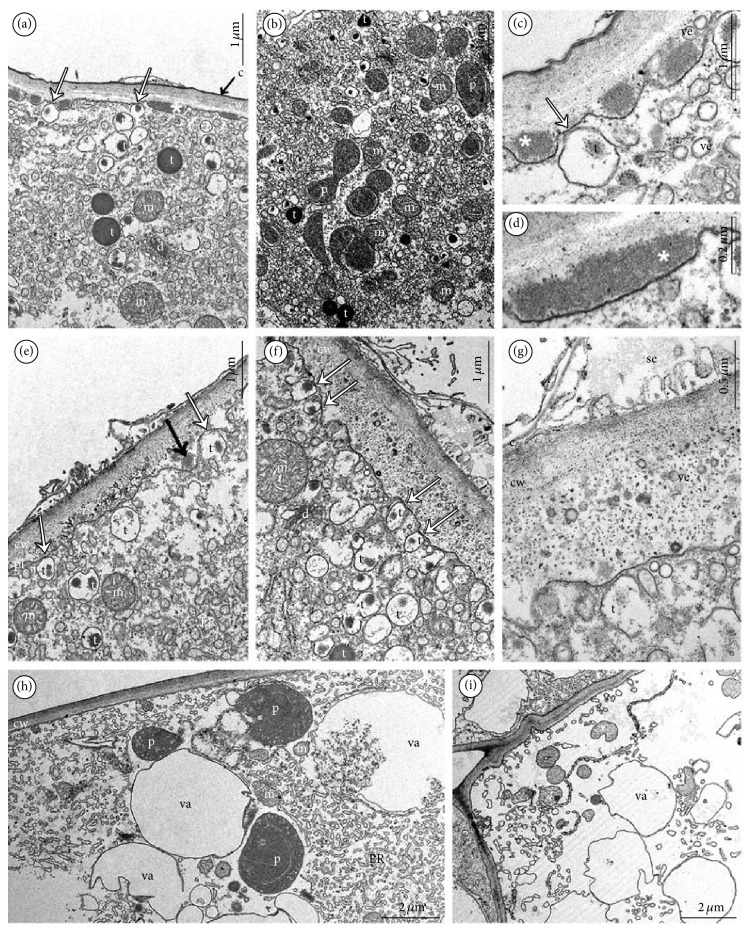
Ultrastructure of papillae (TEM). (a) Dense cytoplasm with profuse rough or smooth endoplasmic reticulum, plentiful vesicles (bigger ones with tannin-like materials), numerous mitochondria, and dictyosomes. Notice the vesicles with tannin-like materials in contact with plasmalemma (*white arrows*) and structurally the same electron-dense bodies (*asterisks*) in cell wall. The thin layer of swelled cuticle (*black arrow*). (b) Dense cytoplasm of papillae with spherical mitochondria, plastids with tubular structures, and tannin-like materials. (c) Magnification of (a), vesicle with tannin-like materials in contact with plasmalemma (*white arrows*), the electron-dense bodies (*asterisk*) in cell wall, thin layer of cuticle, small vesicles present in cytoplasm and in cell wall. (d) Magnification of (a), the electron-dense body (*asterisk*) in cell wall. (e, f) In cytoplasm plentiful vesicles are present. (bigger ones with tannin-like materials). Notice the vesicles with tannin-like materials in contact with plasmalemma (*white arrows*) and structurally the same electron-dense body (*black arrow*) after building into plasmalemma. The ruptured, thin layer of swelled cuticle caused by pressure of gathered substances. (g) Cell wall structure with visible numerous vesicles and secretion gathered under the cuticle. (h, i) More vacuolated (va: vacuole) cells of outgrowth under the papillae with starchless plastids with tubular structures (c: cuticle, cw: cell wall, d: dictyosomes, ER: profiles of endoplasmic reticulum, m: spherical mitochondria, p: plastids, pl: plasmalemma, se: secretion, t: tannin-like materials, and ve: vesicles).

On the cross-section of a lip, the cells of furrow were often collapsed (Figures 2(b), 3(a), and 3(c)) and intensively stained in water-insoluble polysaccharides (Figures 3(a) and 3(c)) and proteins (not illustrated). The 3–5 vascular bundles were noted in parenchyma under the furrow and under the outgrowths with papillate ridges (Figure 3(a)). The tiny starch grains were noted in all lip cells, especially close to the vascular bundles of outgrowths forming ridges with papillae (Figures 3(a) and 3(b)). The presence of starch was various in flowers, without regularity. At the end of anthesis, the starch grains were visible only in outer (abaxial) lip epidermis (Figure 3(d)). The exudation on papillae (Figure 3(e)) was noted through the whole anthesis and was stained on proteins (Figure 3(f)) and lipids (not illustrated). During the secretion process, the enlarged cell nuclei were observed. The staining with Auramina O revealed that the cuticle was very thin (Figure 3(g)). The tissue of other tepals was homogeneous (Figure 3(h)) and was stained on proteins (Figure 3(i)) with tiny starch grains (Figure 3(j)) and with no secretion on their surface. TEM studies of papillae displayed features of secretory activity. The dense cytoplasm contained profuse profiles of rough or smooth endoplasmic reticulum (ER), plentiful vesicles (bigger ones with tannin-like materials), and numerous spherical mitochondria, sometimes dictyosomes and starchless plastids with tubular structures (Figures 4(a), 4(b), 4(e), and 4(f)). The vesicles with tannin-like materials were in contact with plasmalemma (Figures 4(a), 4(c), 4(e), and 4(f)). The electron-dense bodies visible in cell wall structurally resembled the tannin-like material. Moreover, the small vesicles were visible close to irregular plasmalemma and in the cell wall structure (Figures 4(c), 4(e), and 4(f)). The secretion was gathered underneath the thin cuticle (Figures 4(a), 4(e), 4(f), and 4(g)) and caused its rupture (Figures 4(e) and 4(f)). The cells of the outgrowth under the papillae were more vacuolated than papillate cells (Figures 4(g) and 4(h)). Like in papillae (Figure 4(b)), the starchless plastids with tubular structures were visible also in these cells (Figure 4(h)).

**Figure 5:**
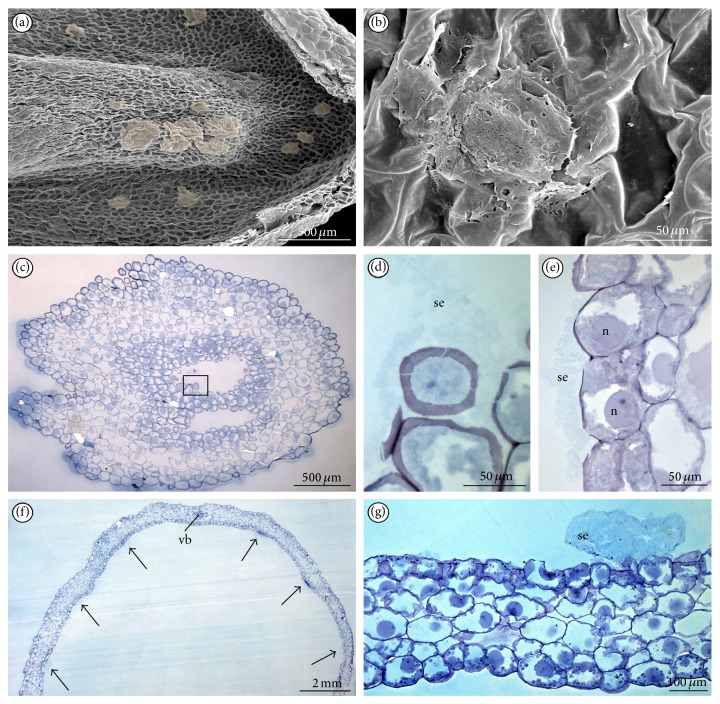
Lip spur, 4th day of anthesis: (a) surface of adaxial (inner) part of spur with places of secretion (*yellow*) (SEM). (b) Secretion in the apex of the spur (SEM). (c) Cross-section of spur, the apex of the spur with secretion (TBO). (d) Magnification of (c), secretion (TBO). (e) Further part of spur with visible secretion, large nuclei in dense cytoplasm (TBO). (f) Cross-section of the middle part of spur, the places with exudation (*arrows*) (TBO). (g) Large amount of the secretion inside spur, stained for proteins (ABB) (se: secretion).

**Figure 6:**
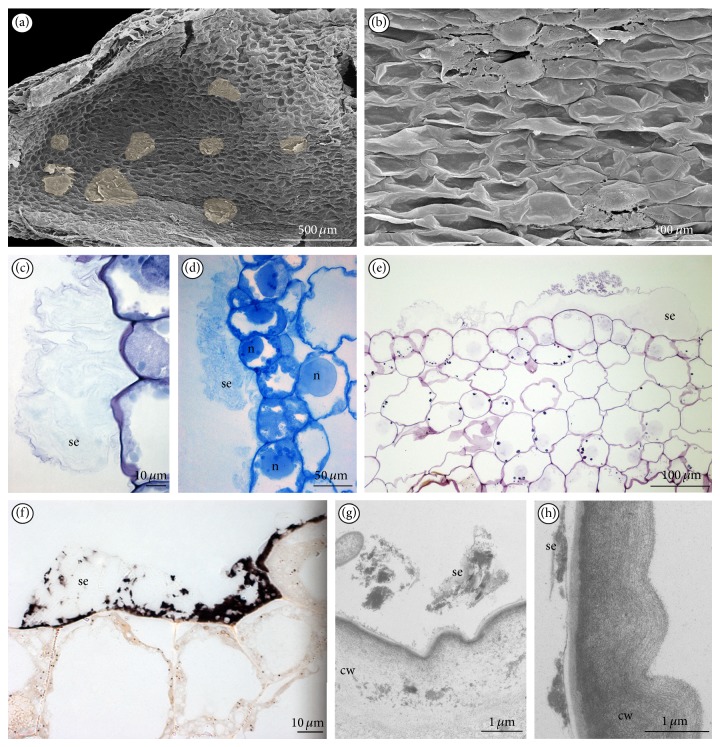
Lip spur: (a) adaxial (inner) surface of spur with places of secretion (*yellow*) at the apex of the spur, the 6th day of anthesis (SEM). (b) Places of secretion inside spur, 4th day (SEM). (c) Secretion on the adaxial surface of spur, 2nd day (TBO). (d) Secretion on the adaxial surface of spur stained for proteins; notice large nuclei, 2nd day (ABB). (e) Secretion inside spur stained for polysaccharides insoluble in water, starch grains visible in cells, 5th day (PAS). (f) Secretion on the adaxial surface of spur, stained for lipids (SBB). (g-h) Secretion on the adaxial surface of spur (TEM) (cw: cell wall; se: secretion).

The SEM results and the cross-sections of spur from different days revealed that the exudation was irregularly present in few places along its whole length (Figures 5(a), 5(b), 6(a), and 6(b)). The secretion was noted on some cells of adaxial (inner) epidermis along the entire length of spur (Figures 5(c), 5(d), 5(e), 5(f), and 5(g)), but in a larger amount at the apex of spur (Figures 5(c) and 5(d)). Though the spur was in the upper position, the nectar was not dripping down. The exudation (Figure 6(c)) was stained on proteins (Figure 6(d)) and slightly on water-insoluble polysaccharides (Figure 6(e), compared with labellar papillae Figures 3(b), 3(e), and 3(f)). The enlarged nuclei were visible in secretory cells (Figures 5(e) and 6(d)), whereas the nuclei were significantly smaller after cessation of secretion.

The idioblasts with raphides were present mainly in tepals tissue (Figure 3(h)) and sporadically in lip and spur tissues.

## 4. Discussion

The morphological, histochemical, and ultrastructural studies of flowers collected at different days of anthesis provide strong evidence to conclude that nectar is secreted on the upper surface of papillae on lip ridges and adaxial epidermis in spur of* Epipogium aphyllum*. Nectar-secreting epidermal structures, such as trichomes, papillae, or glands, are relatively common in plants [21], for example, the nectary trichomes of* Abutilon pictum* or nectary papillae of* Lonicera japonica* [22]. The nectar present on papillae and exuded irregularly trace nectar in spur in* Epipogium aphyllum* are available for visiting insects, such as* Meliscaeva cinctella* or* Chrysanthia geniculata* and also for pollinators,* Bombus*.* Meliscaeva cinctella* was also recorded as licking the nectar from lip and inside spur without taking pollinaria in Austrian population of* Epipogium aphyllum* [2]. The papillae on outgrowths forming ridges were supplied by collateral vascular bundles. With the highest probability, sugar and water ingredients of nectar are delivered to nectariferous cells through sieve tubes [23–26]. In* Hexisea imbricata*, sugar deposited as starch, as in the nectary cells of* Epipogium aphyllum*, is transported through the phloem elements of collateral bundles [27]. During secretion, the starch is hydrolyzed and is not found in plastids, as in plastids of* E. aphyllum*. The starch is regarded as sugar source for the nectar production or source of energy for metabolic processes (e.g., [27–32]).

Some typical features of nectariferous cells are noted in flowers of* Epipogium aphyllum* [33]: highly enlarged nuclei, many profiles of rough and smooth endoplasmic reticulum, starch grains, and numerous vesicles immersed in a dense cytoplasm. The secretory cells are smaller than parenchymatous cells, like in* Limodorum abortivum* [34]. The sugars are transported in ER and after fusion with plasmalemma, they are released to the external surface (model proposed by [35], confirmed by [20, 27, 28, 36, 37]). Furthermore, the vesicles fusing with plasmalemma were described as active in granulocrine secretion in other orchid species, that is, in* Restrepia* [36],* Gymnadenia* [38],* Anacamptis* [18],* Bulbophyllum* [19], and* Epipactis* [20]. Under the force of collected nectar, the impermeable cuticle becomes very thin and breaks releasing the nectar outward [33, 39]. The second route to secrete nectar is diffusion through tiny secretory cell walls [40]. Nectar could be also exuded through modified stomata (i.e., [41]), which was not observed in the* Epipogium aphyllum*.

The occurrence of large quantities of tannin-like materials in vacuoles is confined to papillae, as in other mycoheterotrophic plants, Monotropaceae, where tannins (and also pigments) are restricted to epidermal cells ([42] after [43]). In our opinion, because of the same ultrastructural appearance, the large electron-dense bodies gathered close to anticlinal cell walls are tannin-like materials. The very similar structures, but in larger quantities and occurring in anticlinal and periclinal cell walls, have been investigated in* Musa paradisiaca *var.* sapientum* [22]. Tannins function as a protective barrier against herbivores, pathogens, and UV radiation. The insects licking the secretion accumulated on papillae on lip of* Epipogium aphyllum* are probably deterred from tissue consumption. Tannins are also noted in calluses or cell suspension cultures [44–46] and in petals [47], increasing in organelles called tannosomes [48]. The noticeable is the fact that idioblasts with raphides sporadically appear in lip and spur tissues, but more often in other tepals tissue. The role of calcium oxalate crystals aggregated in raphides consists also, like that of tannins, in deterring the herbivores [49]. Reassuming, the tannin-like materials are mainly in lip tissue, whereas raphides are mainly in other tepals, the same as in* Bulbophyllum wendlandianum* [19]. Therefore, in our view, the protection against herbivores is regulated by both tannins and calcium oxalate crystals, which can be produced in tissues in different quantities. Large quantities of tannin-like materials in lip tissue can be explained by the fact that beetles, the main pollinators, are clumsy and destructive for flowers. They feed on nectar, pollen, and floral tissue; find shelter for prolonged periods in flowers; or congregate for mating purposes [50].

Comparing the features of trace nectar present on papillae and inside spur in the* Epipogium aphyllum* with other mycoheterotrophic orchid* Limodorum abortivum* with spur functioning as a nectar reservoir [34], we did not record similarities such as abundance of dictyosomes and presence of lipid bodies and plastoglobuli in plastids. Dictyosomes (abundant in* L. abortivum*, few in* Epipogium aphyllum*) and ER (profuse in both species) are involved in nectar transport. Although dictyosomes are commonly less abundant in cytoplasm at the secretory stage [36], as was seen in* E. aphyllum*, lipid bodies were frequently recorded in nectaries [28, 34, 37, 38, 51] and osmophores [18, 36, 52, 53]. In* Epipogium aphyllum*, the lipid bodies are not observed in cells; however, the secretion on the surface was staining on lipids. Such lipid surface layer plays a role in reduction of water evaporation and lipids may be also a reward for pollinators [54]. The cell wall ingrowths are not observed in nectariferous cells of* E. aphyllum*, as in* Epipactis palustris* [20] and* Limodorum abortivum *[29]. This is explained by nectar chemistry [55]. The exuded nectar with the dominance of sucrose (in* Epipogium aphyllum*, major constituents are disaccharides [3], probably sucrose) and not hexose, is characterized by the absence of ingrowths in cell walls. The plastids with plastoglobuli (also called osmiophilic bodies) occur in osmophores and nectaries [28, 34, 38, 56], but not in plastids of* E. aphyllum*.

## 5. Conclusion 

The Ghost Orchid is fascinating because of its ecological behavior. Additionally, the floral biology and ultrastructure differ from mycoheterotrophic plants known up to date. In this study we have proved that (1) flowers of* Epipogium aphyllum* secrete nectar, (2) nectar is produced in two sites: on papillae of the lip and inside spur independently, (3) the secretory tissue is responsible for secretion (endocrine secretion) both on lip and in spur, (4) the dynamic changes of the nectar exudation are noticeable during the anthesis: both on the lip and inside the spur, and (5) nectar secretion is not dependent on the colour of* E. aphyllum* blooming shoots.
